# Tobacco smoking in non-psychotic patients with suicidal ideation

**DOI:** 10.1192/j.eurpsy.2021.1574

**Published:** 2021-08-13

**Authors:** M. Zinchuk, G. Kustov, M. Beghi, E. Pashnin, A. Yakovlev, A. Avedisova, A. Guekht

**Affiliations:** 1 Suicide Research And Prevention, Moscow Research and Clinical Center for Neuropsychiatry, Moscow, Russian Federation; 2 Department Of Mental Health, AUSL Romagna, Cesena, Italy; 3 Functional Biochemistry Of The Nervous System Lab, Institute of Higher Nervous Activity and Neurophysiology of RAS, Moscow, Russian Federation; 4 Therapy Of Mental & Behaviour Disorders Department, National Medical Research Centre for Psychiatry and Narcology by Serbsky V, Moscow, Russian Federation; 5 Mental And Neurological Disorders Department, Moscow Research and Clinical Center for Neuropsychiatry, Moscow, Russian Federation

**Keywords:** Suicide, NSSI, Tobacco smoking, Ideation-to-action framework

## Abstract

**Introduction:**

Tobacco smoking (TS) is a major public health concern worldwide because of its association with a number of unfavorable health-related outcomes. According to recent studies TS negatively affects both physical and mental health. Suicidal ideation (SI) is more prevalent in people with mental disorders than in the general population. Factors associated with the transition from SI to suicide attempt (SA) should be detected to prevent suicide in this high-risk population.

**Objectives:**

The aim of the study is to evaluate the influence of tobacco smoking on risk of lifetime suicide plan (SP), SA and nonsuicidal self-injury (NSSI) in patients with nonpsychotic mental disorders (NPMD) and SI.

**Methods:**

Four hundred and 78 consecutive patients with NPMD and SI were included into the study. All patients were evaluated by a psychiatrist, underwent Self-Injurious Thoughts and Behavior Interview as well as semi-structured interview designed to gather information on demographic and biographical features. Mann-Whitney, Fishers exact test, chi-square test and stepwise logistic regression were used as statistical methods.

**Results:**

Three hundred and 24 (67.8%) patients have smoked at least 100 cigarettes in their entire life. No differences were found between smokers and non-smokers in terms of age, gender, educational and occupational statuses as well as age at onset of self-injurious thoughts and behavior, and total number of SP, SA and NSSI (all: p>0.05). The lifetime smokers were at higher risk of SA (OR=2.379; 95% CI 1.58-3.581: p<0.001) and NSSI (OR=1.591; 95% CI 1.064-2.38: p=0.024).

**Conclusions:**

Lifetime smoking in patients with NPMD and SI is associated with SA and NSSI.

